# Hypoxia Induced Impairment of NK Cell Cytotoxicity against Multiple Myeloma Can Be Overcome by IL-2 Activation of the NK Cells

**DOI:** 10.1371/journal.pone.0064835

**Published:** 2013-05-28

**Authors:** Subhashis Sarkar, Wilfred T. V. Germeraad, Kasper M. A. Rouschop, Elisabeth M. P. Steeghs, Michel van Gelder, Gerard M. J. Bos, Lotte Wieten

**Affiliations:** 1 Department of Internal Medicine, Division of Hematology, Maastricht University Medical Center+, Maastricht, The Netherlands; 2 Department of Radiation Oncology (Maastro Lab), GROW School for Oncology and Developmental Biology, Maastricht University Medical Center+, Maastricht, The Netherlands; 3 Department of Transplantation Immunology, Maastricht University Medical Center+, Maastricht, The Netherlands; Karolinska Institutet, Sweden

## Abstract

**Background:**

Multiple Myeloma (MM) is an incurable plasma cell malignancy residing within the bone marrow (BM). We aim to develop allogeneic Natural Killer (NK) cell immunotherapy for MM. As the BM contains hypoxic regions and the tumor environment can be immunosuppressive, we hypothesized that hypoxia inhibits NK cell anti-MM responses.

**Methods:**

NK cells were isolated from healthy donors by negative selection and NK cell function and phenotype were examined at oxygen levels representative of hypoxic BM using flowcytometry. Additionally, NK cells were activated with IL-2 to enhance NK cell cytotoxicity under hypoxia.

**Results:**

Hypoxia reduced NK cell killing of MM cell lines in an oxygen dependent manner. Under hypoxia, NK cells maintained their ability to degranulate in response to target cells, though, the percentage of degranulating NK cells was slightly reduced. Adaptation of NK- or MM cells to hypoxia was not required, hence, the oxygen level during the killing process was critical. Hypoxia did not alter surface expression of NK cell ligands (HLA-ABC, -E, MICA/B and ULBP1-2) and receptors (KIR, NKG2A/C, DNAM-1, NCRs and 2B4). It did, however, decrease expression of the activating NKG2D receptor and of intracellular perforin and granzyme B. Pre-activation of NK cells by IL-2 abrogated the detrimental effects of hypoxia and increased NKG2D expression. This emphasized that activated NK cells can mediate anti-MM effects, even under hypoxic conditions.

**Conclusions:**

Hypoxia abolishes the killing potential of NK cells against multiple myeloma, which can be restored by IL-2 activation. Our study shows that for the design of NK cell-based immunotherapy it is necessary to study biological interactions between NK- and tumor cells also under hypoxic conditions.

## Introduction

Multiple myeloma (MM) is an incurable plasma cell malignancy that is usually localized within the bone marrow (BM) [1]. Cellular immunotherapy could be an interesting novel treatment option for MM. Natural killer (NK) cell based therapies are of special interest since NK cells and even alloreactive NK cells, can be given to patients without causing graft versus host disease (GvHD). This is in contrast to alloreactive T cells and therefore an important benefit above the procedures focusing on T cell activity. In *in vitro* MM models, NK cells, both autologous and allogeneic, have been demonstrated to effectively eliminate MM cells [2], [3]. Furthermore, NK cells isolated from myeloma patients have been expanded with 500 IU/ml IL-2 and shown to have *ex vivo* killing potential against autologous tumor cells [4]. Moreover, patient derived NK cells have been primed *ex vivo* with the tumor cell line CTV-1a resulting in improved killing of autologous and allogeneic MM cells [5]. Garg et al. have demonstrated expansion of patient derived NK cells by K562 cells transfected with 41BBL and membrane-bound interleukin-15 in the presence of 300 U/mL IL-2 [6]. More importantly, these expanded NK cells reduced myeloma burden in immunodeficient mice, and expanded *in vivo* in an IL-2 dependent fashion. Benson et al. has demonstrated that NK cells derived from MM patients express the inhibitory receptor PD-1 while NK cells from healthy individuals do not express this receptor unless activated by IL-2. They also show that blocking the interaction of the receptor and its ligand PD-L1 increases NK cell cytotoxicity against MM [7]. More recently, anti-KIR antibodies, with the scope of mimicking a KIR-HLA mismatched alloreactive response, have been suggested to provide an alternative strategy to boost NK cell immunity [8]. A first clinical study has shown that administration of IL-2 activated haploidentical KIR ligand mismatched NK cells to MM patients was safe, and 50% of the patients had near complete remission [3]. Together these data show the potential of NK cells in MM and they emphasize that there is room for improvement of the response. Better understanding of the factors influencing effective NK cell anti-tumor responses can help to maximize NK cell anti-MM responses.

The tumor micro-environment can influence disease progression and response to therapy in cancer. Hypoxia is a prominent feature of the tumor microenvironment and considered an adverse prognostic factor best documented for solid tumors [9]. Hypoxia is also a physiological characteristic of the BM [10] and, as shown in mice studies, extremely hypoxic niches are essential for regulating the maintenance and functioning of hematopoietic stem cells [11], [12]. Several recent studies have demonstrated that MM displays features of hypoxia; in the 5T33M mouse MM model, myelomatous BM has been shown to be more hypoxic than normal BM. This was visualized by positive staining of MM BM, for both exogenous- (pimonidazole) and endogenous- (HIF-1α) markers of hypoxia, while normal BM stained only weakly positive [13], [14]. In human BM aspirates, median oxygen tension did not clearly differ between controls and MM patients (around 55 mmHg in all cohorts) [15]. By immunostaining of bone biopsies from the MM patients, this study also showed the accumulation of the hypoxia regulated factor HIF-1α in MM BM, an observation that was in line with two other studies showing the expression of HIF-1α in bone biopsies from MM patients [16], [17]. The accumulation of HIF-1α was indicative of the presence of hypoxic niches in the human BM.

It is now well known that hypoxia contributes to chemo- and radiotherapy resistance of tumor cells [18]. By contrast, our understanding on how hypoxia assists tumor cells in escaping from immune-surveillance is in its infancy, but, increased knowledge could help to make immunotherapy more effective. One reported mechanism of tumor cell escape is hypoxia-induced shedding- and decreased surface expression of MHC class I chain-related (MIC) molecules resulting in reduced cytotoxicity of IL-2 stimulated peripheral blood lymphocytes (PBL) against prostate cancer cells [19], [20]. The impact of hypoxia on NK cell function has been examined in only a very limited number of studies; in a first study, mouse YAC-1 cells were lysed at 21% and 1% oxygen, but were moderately killed by NK cells at 0% oxygen [21]. By contrast, a second study described a decrease, at 2% and 1% of oxygen, in NK cell killing of the K562 cell line [22], the human MHC negative equivalent of mouse YAC-1. The latter study also showed a partial reduction of NK cell cytotoxicity against human liver tumor cell lines at low levels of oxygen. To investigate if hypoxia is an inhibitory factor for NK cell immunity against hematological cancers, these first, partially contradictory, findings need to be further investigated in HLA expressing hematological cells.

In the present study, we hypothesize that one of the biological reasons for limited clinical success of NK cell therapy is, that suppressive factors, like hypoxia, in the BM environment decrease NK cell anti-MM responses. To study this hypothesis, we aim to investigate the influence of hypoxia on NK cell anti-MM responses using *in vitro* approaches where oxygen levels are representative of the tumor micro-environment. Secondly, we are interested in the impact of hypoxia on NK cell activating and inhibitory receptors in attempt to unravel the mechanism at play, as a disturbance by hypoxia on these balancing signals could be limiting effective NK cell therapy.

## Materials and Methods

### Cell lines and culture systems

K562 (obtained from the ATCC) and the myeloma cell lines RPMI-8226/S (obtained from ATCC), OPM-1 and L-363 (received from Dr. A. Martens, UMC Utrecht, The Netherlands) [23] were cultured in RPMI-1640 medium (Gibco, Breda, The Netherlands) supplemented with 10% fetal calf serum (Integro, Zaandam, The Netherlands), 100 U/mL penicillin (Gibco) and 100 µg/mL streptomycin (Gibco) at 37°C in humidified air containing 5% CO_2_. NK cells were isolated from buffy coats by density gradient centrifugation and negative selection of NK cells using MACS beads and columns according to manufacturer’s protocol (Miltenyi Biotech, GmbH, Bergisch Gladbach, Germany). The use of buffy coats, being a side product from an Medical Ethical Review Committee (METC) required procedure, does not need ethical approval in the Netherlands, under the Dutch Code for Proper Secondary Use of Human Tissue. These buffy coats were anonymous, and the individuals from whom the samples originated did not object to their use. MACS sorted human NK cells were cultured in RPMI-1640 medium (Gibco) supplemented with 10% fetal calf serum (Integro), 100 U/mL penicillin (Gibco) and 100 µg/mL streptomycin (Gibco) at 37°C in humidified air containing 5% CO_2_ and at 0% O_2_, 0.2% O_2_, 1% O_2_ or 21% O_2_. For experiments with IL-2 activation, NK cells were activated with 1000 IU/ml recombinant human IL-2 (Proleukin, Novartis) for 14–16 hours at 21% O_2_ or 0% O_2_. After 16 hours, the activated NK cells were resuspended in their culture plates and directly used for cytotoxicity and CD107a assays, without any wash step. *In vitro* hypoxia experiments were performed using a hypoxia chamber system at 0% O_2_ (MG*500*-Don Whitley Scientific Ltd, UK), 0.2% O_2_ and 1% O_2_ (Invivo_2,_ 1000 Ruskinn Technology Ltd, Bridgend, UK) at 37°C with 5% CO_2_.

### Flowcytometry

MM cell ligand expression on OPM-1 and NK cell receptor, perforin and granzyme B expression on freshly obtained NK cells was determined after 14–16 hours incubation at 21% or 0% O_2_. For surface staining, cells were washed with FACS buffer (PBS, 1% FCS) and stained for 30 minutes on ice in the dark. Intracellular staining (perforin/granzyme B) was performed using the cytofix/cytoperm kit (BD Pharmingen, Erembodegem, Belgium) according to manufacturer’s protocol. Antibodies for MM cell ligands and NK cell receptors are listed in Table S1. Flow cytometric analyses were performed with BD FACS Canto II. Data were analyzed with FlowJo 7.6 or BD FACSDiva Software v6.1.2.

### Viability assay

The PE-Annexin V Apoptosis Detection Kit I (BD Pharmingen) was used for detection of apoptotic NK cells. After 14 to 16 hours incubation at 21% or 0% O_2_, cells were washed with PBS (Sigma-Aldrich, Zwijndrecht, the Netherlands) and stained with 7-AAD and Annexin V-PE. After 15 minutes incubation in the dark at room temperature, samples were analyzed with a BD FACS Canto II. Cell viability of MM was estimated at 21% or 0% O_2_ using propidium iodide (PI).

### Cytotoxicity assay

Cytotoxic potential of NK cells was determined in a 4.5 hour flow cytometry based assay. Effector NK cells were isolated from buffy coats of healthy individuals by negative selection on MACS columns (Miltenyi Biotech). Target cells were labeled with DiO (Sigma-Aldrich). Both effector and target cells were individually pre-incubated for 14–16 hours at 21% or 0-1% O_2_ and subsequently combined in triplicate at E:T ratios 5∶1, 10∶1 and 20∶1 in Corning®Costar® round-bottom 96 well plates. After 4.5 hours, cell death of DiO positive target cells was measured with PI and specific cytotoxicity was determined by the equation: (% PI positive target cells − % spontaneous PI positive cells)/(100 - % spontaneous PI positive cells) ×100.

### CD107a assay

Degranulation of cytotoxic contents from NK cells was measured by analysis of the degranulation marker CD107a by flow cytometry [24]. MACS enriched NK cells and OPM-1 or K562 cells were individually pre-incubated for 14–16 hours at 21% or 0% O_2_ and thereafter combined at a 2∶1 E:T ratio at either 21% or 0% O_2_ for 4.5 hours. APC labeled anti-CD107a (clone: H4A3, BD Pharmingen) or the corresponding isotype was added to the wells within 5–10 minutes after combining NK and OPM-1 cells. As a positive control PMA (100 ng/ml, Sigma-Aldrich) and ionomycin (1 µg/ml; Sigma-Aldrich) were added to the NK cells during the 4.5 hours degranulation assay.

### NKG2D blocking

Anti-NKG2D antibody (clone 1D11) or matched IgG1 isotype control, both LEAF purified and obtained from Biolegend (San Diego, CA, USA), at a final concentration 10 µg/ml were added to NK cells 20 minutes before combining it with OPM-1 cells at E:T ratio 20∶1 in a 4.5 hour cytotoxicity assay.

### Statistical analyses

Statistics were performed with GraphPad Prism V (Graphpad Software Inc, San Diego, CA, USA). Effect of 0% O_2_ on surface receptor expression was analyzed by paired *t*-test. Influence of hypoxia on NK cytotoxicity and degranulation was evaluated with repeated measures ANOVA with Bonferroni correction. *p<0.05 was considered statistically significant.

## Results

### NK cell cytotoxicity towards MM cells is decreased during hypoxia

**Figure 1:**
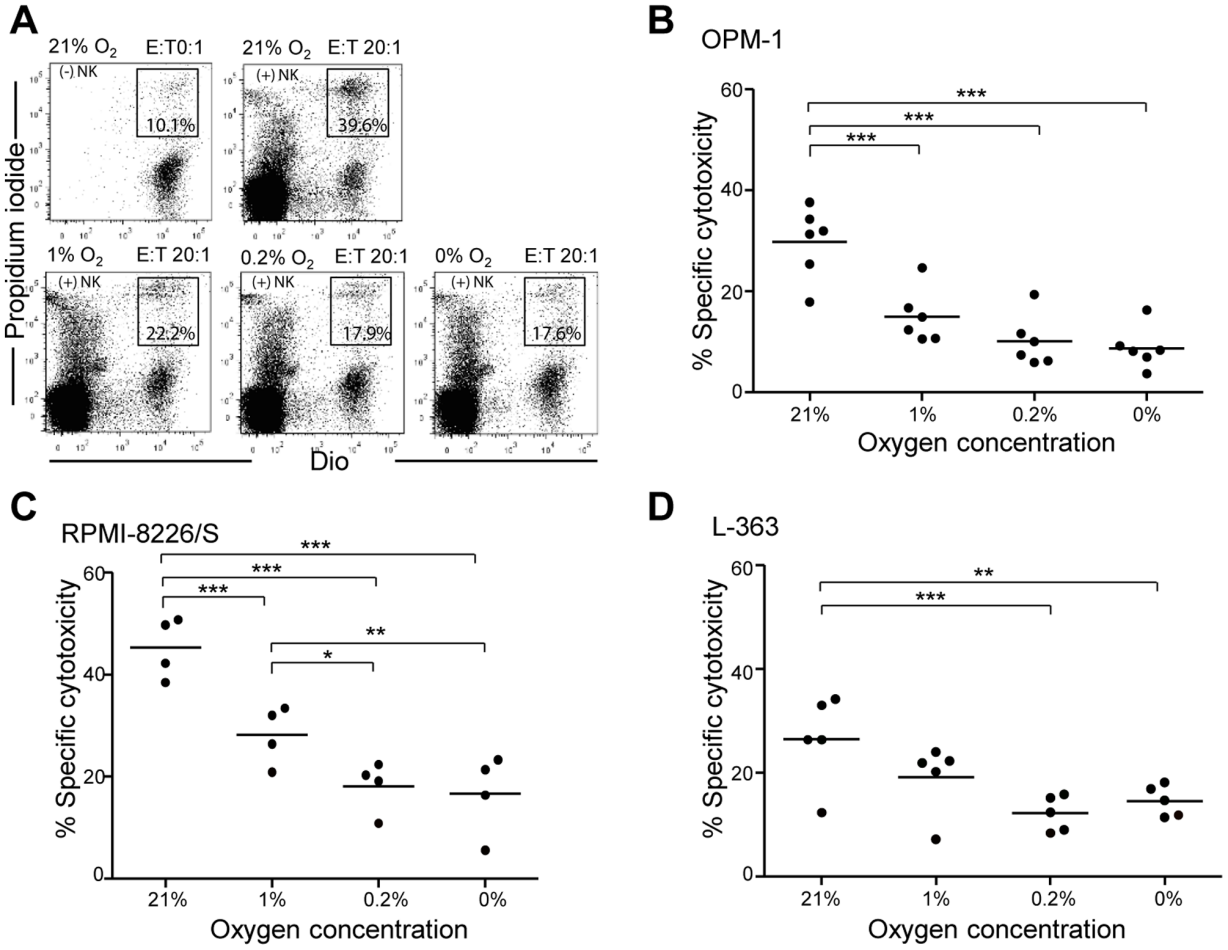
Hypoxia diminishes the cytotoxic potential of NK cells. Freshly isolated NK cells and DiO labeled myeloma cell lines (OPM-1, RPMI-8226/S and L-363) were cultured at 21%, 1%, 0.2% or 0% O_2_, for 14–16 hours, followed by assessment of the cytotoxic potential of NK cells in a 4.5 hour assay at corresponding oxygen levels. (A) Representative dotplots of OPM-1 cells cultured with NK cells at 20∶1 (E:T) ratio and (B–D) quantification of dotplots of 4–6 donors for different MM cell lines. Each dot represents mean of triplicate cultures of an individual donor. Statistics were performed with one-way repeated measures ANOVA with Bonferroni correction * *p*<0.05, ** *p*<0.01, *** *p*<0.001.

Oxygen levels within solid tumors can be as low as 0% O_2_ [25], and oxygen levels ≤1% O_2_ have been reported in the bone marrow [26] where MM cells reside [15]. Furthermore, high metabolic rates of proliferating MM cells presumably add to the creation of a hypoxic tumor micro-environment. As we intend to develop allogeneic NK cell therapy for this disease it is crucial to study NK cell mediated anti-MM responses at low levels of oxygen. We thus examined NK cell cytotoxicity by pre-incubating MM cell lines and NK cells at different oxygen levels, followed by assessment of cytotoxicity. This revealed that specific NK cell cytotoxicity was decreased at 1%, 0.2% and 0% compared to 21% O_2_ in all three of the tested MM cell lines (OPM-1, RPMI-8226/S and L-363) (Figure 1A–D). This observation was also confirmed at lower E:T ratio of 5∶1 and 10∶1 for the OPM-1 cell line (Figure S1). The decreased cytotoxicity under hypoxia was unrelated to NK cell viability because the percentage of viable NK cells (negative for Annexin V and 7AAD), after 16 hours of incubation at 0% or 21% O_2_, was comparable. (Figure S2A–B) In addition, hypoxia exposure did not induce death of MM cells (Figure S2C–D).

### Exposure to hypoxia decreases surface expression of NKG2D and CD16 on NK cells

**Figure 2:**
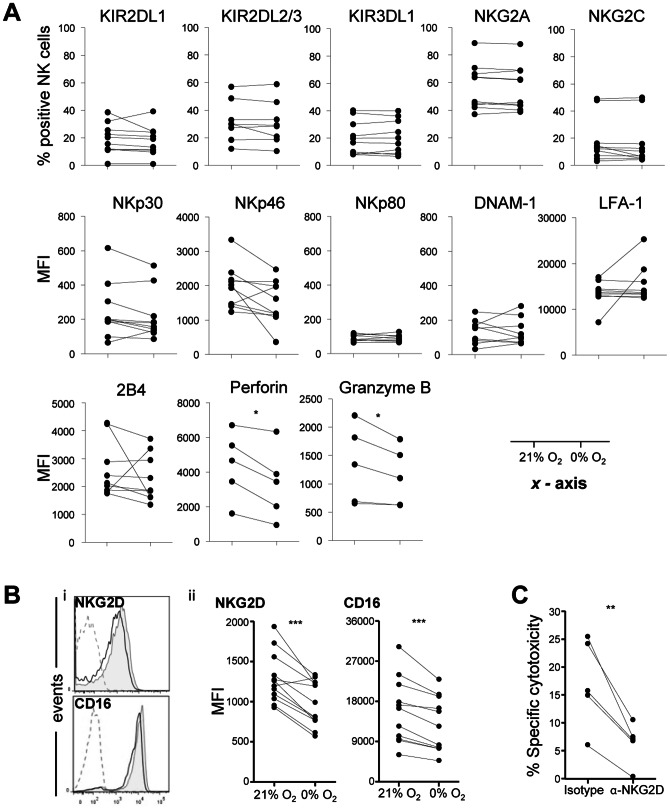
Hypoxia reduces surface expression of NKG2D and CD16 on NK cells and intracellular perforin and granzyme B. (A) Flow cytometric analysis of surface expression of NK cell receptors and intracellular perforin and granzyme B after 14–16 hours of incubation at 21% or 0% O_2_. Graphs depict quantification of the % positive NK cells or MFI for the different receptors. Each dot represents one donor. Shown alongside the graphs is the *x*-axis oxygen parameter for the graphs. (B) Flow cytometric analysis of surface expression of NKG2D (n = 12) and CD16 (n = 11) on NK cells after 14–16 hours of incubation at 21% or 0% O_2_. (i) Representative histograms of surface expression at 21% O_2_ (grey filled) or 0% O_2_ (solid black line) and matched isotype control (dashed line) are displayed. (ii) Graphs represent quantification of the independent donors. (C) Cytotoxicity assay at E:T 20∶1 in the presence of 10 µg/ml α-NKG2D blocking antibody or matched isotype control. Each dot represents an individual donor. Statistics were performed with paired *t*- test. ** *p*<0.01 and *** *p*<0.001.

**Figure 3:**
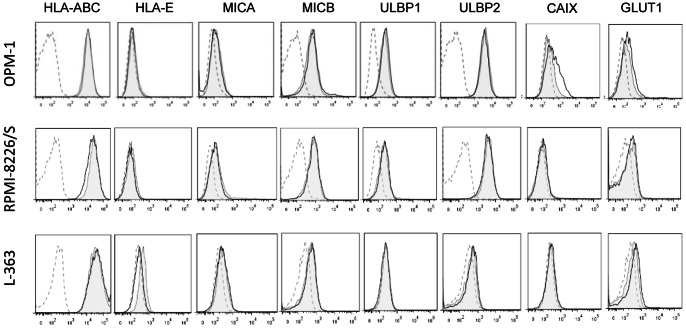
Hypoxia does not have an effect on surface expression of HLA-ABC and –E and NKG2D ligands. HLA-ABC, HLA-E and NKG2D ligands MICA/B and ULBP1-2 surface expression on OPM-1 target cells was analyzed after 14–16 hours of incubation at 21% O_2_ (grey filled) or 0% O_2_ (solid black line). Matched isotype control is depicted by a dashed line. Expression of hypoxia associated hypoxia makers CAIX and GLUT1 was analyzed by flowcytometry after 14–16 hours incubation at 21% O_2_ (grey filled) or 0% O_2_ (solid black line). Matched isotype control is depicted by a dashed line.

NK cell cytotoxicity is regulated by the balance of signaling through inhibitory and activating receptors [27]. As we found a decreased cytotoxicity at low oxygen tension, we examined if hypoxia induced phenotypic changes to NK cells or MM cells. For this, we studied the influence of hypoxia on surface expression of NK cell receptors. Expression levels of inhibitory receptors (KIRs and NKG2A), most activating receptors (NKG2C, NCRs, DNAM-1, and 2B4) and the adhesion molecule LFA-1 on NK cells were not influenced by hypoxia (Figure 2A and S3). By contrast, CD16 (Figure 2B) and intracellular perforin and granzyme B (Figure 2A and S4) expression was slightly but significantly decreased by hypoxia in all donors and NKG2D was lower in 11 of 12 analyzed donors (Figure 2B). NCR, DNAM-1 and NKG2D have been suggested to play a role in activation of NK cells by MM target cells [2]. To confirm that NKG2D was important in our system, we blocked the receptor using a specific antibody. The resulting decrease in killing of target OPM-1 cells at 21% O_2_ showed that NKG2D was indeed important for NK cell cytotoxicity against OPM-1 cells (Figure 2C). In addition to analysis of the receptors, we measured surface expression levels of several NK cell ligands on MM cells upon 16 hours of incubation at 21% and 0% O_2_. HLA–E, the ligand for NKG2A, was not present on the surface of OPM-1 and RPMI-8226/S cells, whereas it was expressed at low levels by L-363 (Figure 3). HLA-ABC, the ligand for inhibitory KIRs was expressed at high levels by all three cell lines. Neither HLA-ABC nor HLA-E levels were clearly influenced by hypoxia. We also analyzed the influence of hypoxia on activating NKG2D ligands. Both OPM-1 and RPMI-8226/S cells expressed very low levels of MICA, intermediate levels of MICB and ULBP1 and somewhat higher levels of ULBP2, and the expression was not influenced by hypoxia. L-363 cells were negative for ULBP1 and had very low expression of MICA, MICB and ULBP2 which did not change upon incubation at low oxygen levels. For OPM-1, the hypoxia associated surface marker GLUT1 was enhanced upon incubation at 0% O_2_. Another hypoxia associated marker, Carbonic anhydrase IX (CA IX) was upregulated on OPM-1 cells confirming the presence of hypoxia during the incubation. On RPMI-8266/S and L-363, GLUT1 and CAIX were not distinctly influenced by hypoxia. Overall, hypoxia had only a small effect on the phenotype of NK- and MM cells, but, possibly a relevant effect on NKG2D and CD16.

### Adaptation of NK cells or MM cells to hypoxia is not required for the hypoxia-induced decrease in NK cell cytotoxicity

**Figure 4:**
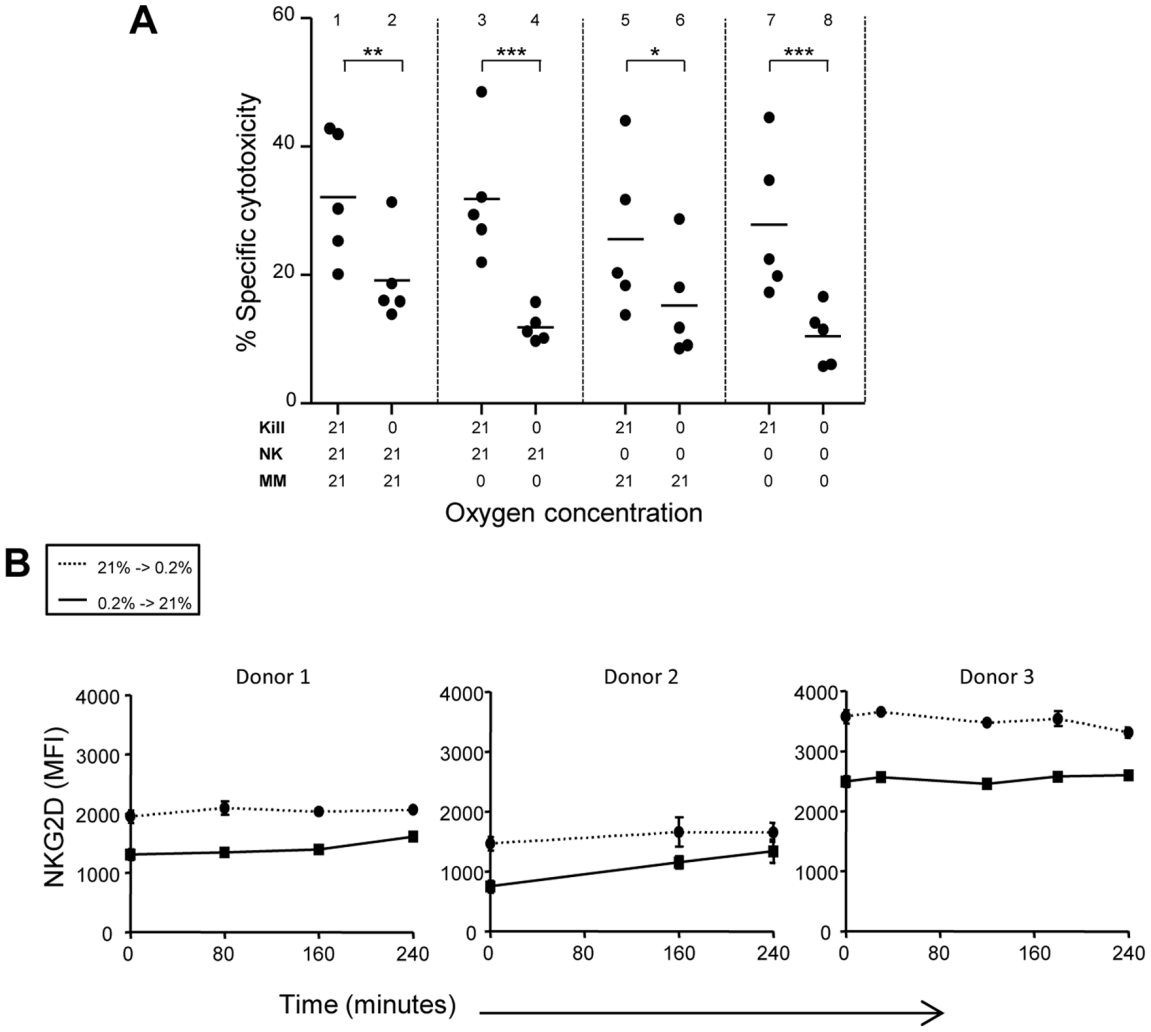
Oxygen concentration during NK cell killing is the key regulating parameter determining the cytotoxic potential. (A) Pre-incubation (16 hours) of MM- and NK cells followed by 4.5 hour assessment of cytotoxicity were performed at the O_2_ concentration depicted on the x-axis. Each dot represents mean of triplicate cultures of individual donors. Statistics were performed with one-way repeated measures ANOVA with Bonferroni correction * *p*<0.05, ** *p*<0.01, *** *p*<0.001. (B) To determine the kinetics of NKG2D downregulation by hypoxia, NK cells were incubated for 16 hours at 21% or 0.2% oxygen. Thereafter, cells were transferred from 21% to 0.2% or from 0.2% to 21% oxygen. After the time indicated on the x-axis, NKG2D expression was measured by flowcytometry. Data show means +SD per donor of triplicate cultures.

To further investigate whether hypoxia-induced changes to either NK- or MM cells, or both, were responsible for the observed decrease in cytotoxicity, we pre-incubated NK cells, MM cells, or both at 21% or 0% O_2_ and combined them for cytotoxicity assessment at 21% or 0% O_2_. Pre-incubation of NK cells or OPM-1 cells at 0% O_2_ did not influence NK cell cytotoxicity (Figure 4A; group 3 and 5). By contrast, all conditions where the cytotoxicity assay was performed at 0% O_2_ (group 2, 4, 6 and 8), showed decreased NK cell cytotoxicity as compared to corresponding conditions with the cytotoxicity assay at 21% O_2_ (Figure 4A; group 1, 3, 5 and 7). Thus, the critical factor proved to be the O_2_ concentration during the 4.5 hour cytotoxicity assay, hence reintroducing O_2_ to tumor or NK cells cultured under hypoxic circumstances restored the cytotoxic effect almost immediately (for example group 7 vs 8). To determine if this could be explained by the hypoxia-induced decrease in NKG2D surface expression levels, we examined the kinetics of hypoxia-induced NKG2D downregulation. In line with the cytotoxicity assay described in figure 4A, we first pre-incubated NK cells for 16 hours at either 21% or 0.2% of O_2_ and subsequently incubated them for another 4 hours at 21% (0.2% pre-incubated cells) or 0.2% (21% pre-incubated cells). For none of the donors the 4 hours of incubation at 0.2% O_2_ reduced the NKG2D expression to the level observed after 16 hours of pre-incubation. Also, for the restoration of NKG2D levels, 4 hours at 21% was not sufficient (Figure 4B).

### NK cell degranulation can occur under hypoxia but the percentage of degranulating NK cells is decreased

**Figure 5:**
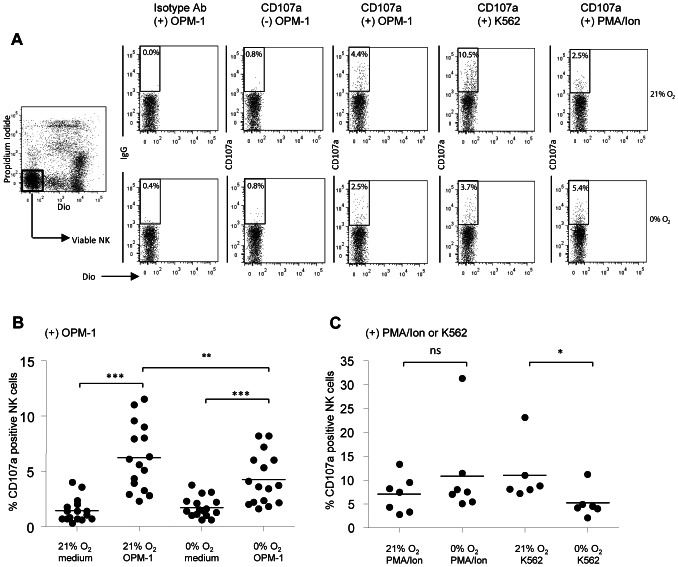
NK cell degranulation can occur under hypoxia but the percentage of degranulating NK cells is decreased. NK cells and target cells (OPM-1 or K562) were pre-incubated for 14–16 hours at 21% or 0% of oxygen before they were combined in a 4.5 hour degranulation assay in the presence of an antibody specific for the degranulation marker CD107a or the corresponding isotype control. Percentage of CD107a positive NK cells for the isotype controls was always less than 0.5%. To some of the cultures PMA/ionomycin was added during the 4.5 hour degranulation assay. The percentage of degranulated, CD107a positive NK cells was subsequently measured by flowcytometry. A) Dot plots depicting data obtained for one representative donor. B) Quantification of dot plots showing the percentage of CD107a positive NK cells upon incubation with medium or OPM-1 target cells. C) Quantification of dot plots showing the percentage of CD107a positive NK cells upon incubation with PMA/ionomycin or K562 target cells. Each dot in B and C represents mean of duplicates of individual donors. Statistics were performed with one-way repeated measures ANOVA with Bonferroni correction * *p*<0.05,** *p*<0.01, *** *p*<0.001.

As adaptation to hypoxia by NK or MM cells was not essential for the hypoxia-induced decrease in killing, we evaluated whether MM cells were able to induce NK cell degranulation under hypoxia. For this, we studied NK cell degranulation by analysis of the specific marker CD107a. We performed this in the absence of monensin to avoid interference with pH regulatory systems of the cell under hypoxia. NK cells did not vigorously degranulate in the absence of MM target cells. However, both at 21% and at 0% of O_2_, addition of OPM-1 target cells to the NK cell cultures resulted in a significantly higher percentage of CD107a positive NK cells as compared to the corresponding condition without OPM-1 (Figure 5A,B). This demonstrated that NK cells maintained the ability to degranulate in response to OPM-1 under hypoxia. However, the percentage of degranulating NK cells in response to OPM-1 at 0% O_2_ was slightly, but, significantly lower than the percentage of degranulating NK cells at 21% O_2_ (Figure 5B and S5). To investigate whether this decrease was caused by an intrinsic defect of NK cells to degranulate under hypoxic conditions we incubated NK cells with the universal control PMA/ionomycin or HLA-negative K562 cells at 21% or 0% O_2_. This revealed that the percentage of CD107a positive NK cells upon PMA/ionomycin stimulation was comparable for 21% and 0% O_2_ (Figure 5A,C). Yet upon incubation with K562 cells, the percentage of degranulating NK cells at 0% O_2_ was lower than the percentage of degranulating NK cells at 21% O_2_ (Figure 5C and S5).

### NK cell activation by IL-2 can restore cytotoxicity against hypoxic MM cells

**Figure 6:**
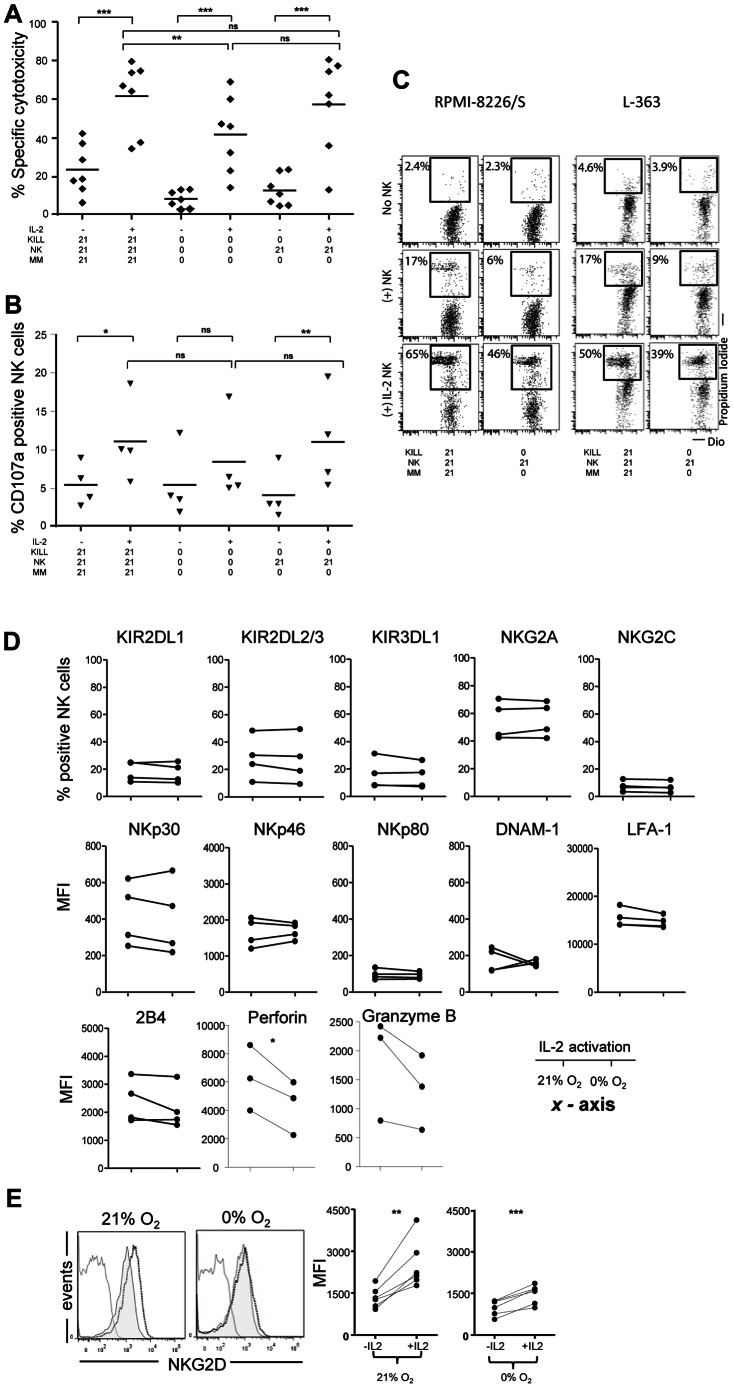
IL-2 activates NK cells as effector cells for targeting multiple myeloma in hypoxic environment. (A–B) Pre-incubation (16h) of NK- and OPM-1 MM cells were performed as indicated on the *x*-axis. IL-2 activation of indicated conditions was done by adding 1000 IU/ml of IL-2 during the 16 hour pre-incubation period. Upon pre-incubation NK cells and MM cells were cocultured in a 4.5 hour cytotoxicty (A) or CD107a (B) assay. In (A) each dot represents mean of triplicate cultures of individual donors. In (B) NK cell degranulation was determined as described in figure 5 by quantification of the percentage of CD107a positive NK cells. Data in (B) show means of duplicate cultures of individual donors. Statistics were performed with one-way repeated measures ANOVA with Bonferroni correction * *p*<0.05, ** *p*<0.01, *** *p*<0.001. (C) NK cells and RPMI-8226/S or L-363 MM cells were pre-incubated as described in 6A and subsequently combined in a 4.5 hour cytotoxicity assay. Plots show data obtained from one donor. (D) Flow cytometric analysis of surface expression of NK cell receptors and intracellular expression of perforin and granzyme B after 14–16 hours of incubation at 21% or 0% O_2_ in the presence of IL-2. Graphs depict quantification of the % positive NK cells or MFI for the different receptors. Each dot represents one donor. Shown alongside the graphs is the *x*-axis oxygen parameter. (E) NKG2D surface expression on NK cells upon 14–16 hours incubation at 21% O_2_ and 0% O_2_ in the presence (solid black line) or absence (grey filled) of IL-2 and matched isotype (grey solid line). Each dot represents an individual donor. Statistics were performed with paired *t*- test. ** *p*<0.01, *** *p*<0.001.

In the context of NK cell immunotherapy, it is important to overcome the adverse effect of hypoxia. Strategies like targeting susceptibility of tumor cells or boosting NK cell cytotoxicity can be proposed. An interesting possibility is *ex vivo* pre-activation of NK cells. Since IL-2 is frequently being applied for clinical application after NK cell infusion [3], [28], [29], we used IL-2 (1000 IU/ml) to activate NK cells *in vitro*. NK cells activated with IL-2 at 21% O_2_, had enhanced killing against OPM-1, both under normoxia and hypoxia (Figure 6A). Activation at 0% O_2_ also boosted the killing potential of NK cells, however to lesser extent than when activated at 21% O_2_. IL-2 activation coincided with increased NK cell degranulation as shown by higher CD107a levels (Figure 6B and S6). That NK cells activated with IL-2 at 21% O_2_ are potent at killing hypoxic myeloma cells was also observed for RPMI-8226/S and L-363 (Figure 6C). Thereby, our data provided proof of concept that pre-activation of NK cells can overcome the inhibitory effects of hypoxia. Drug-induced upregulation of NK cell ligands like MICs and ULBPs is emerging as intervention strategy to improve anti-tumor effects of NK cells by enhancing sensitivity of tumor cells for NK cells [30], [31]. To improve therapy, it could be interesting to combine these strategies with *ex vivo* or *in vivo* activation of NK cells. We therefore studied the influence of hypoxia on surface expression levels of receptors on IL-2 activated NK cells. This revealed that, hypoxia during IL-2 activation did not clearly influence surface expression levels of most NK cell receptors (Figure 6D). By contrast, intracellular perforin and granzyme B were lower expressed in NK cells activated by IL-2 at 0% O_2_ as compared to NK cells activated at 21% O_2_ (Figure 6D). While IL-2 activation enhanced NKG2D expression levels at 21% and 0% O_2_, the effect at 21% was more pronounced than at 0% O_2_ (*p* = 0.02) (Figure 6E).

## Discussion

During disease progression of MM, the BM micro-environment combined with its stromal factors contributes to MM cell survival, outgrowth and drug resistance [32]. Insights into the mechanism of immunosuppression by individual tumor environmental factors can help to improve efficacy of immunotherapy. Despite the well-known presence of hypoxia in the microenvironment of tumors, our understanding of the impact of hypoxia on immune cell function is very limited. With the hypoxic signature in MM being confirmed in recent patient studies [15], [33], we now show that hypoxia is detrimental for NK cell mediated MM cell killing and a factor to consider when designing NK cell based immunotherapy.

In our study, we used a series of MHC class I expressing MM cell lines to more closely mimic the situation in patients because surface expression of MHC class I molecules is an important inhibitory factor for NK cells, and sensitivity of tumor cells for NK cells is regulated by MHC class I. The expression of MHC by our cell lines might also explain the relatively low levels of CD107a that we observed when analyzing NK cell degranulation. The two earlier studies that also used cell lines of hematological origin used MHC class I negative leukemia cell lines that are easy to kill targets, and are frequently being used as positive controls. One of these studies showed that 0% O_2_ but not 1% O_2_ reduced NK cell cytotoxicity against the mouse MHC negative tumor cell line YAC-1 [21]. By contrast, the second study demonstrated that 1% O_2_ was sufficient to reduce NK cell cytotoxicity against K562, the human equivalent of mouse YAC-1 [22]. Our data are in agreement with the latter study and provide first evidence that NK cytotoxicity against MHC positive MM is severely impaired at oxygen levels representative of the BM environment.

In the current study, we focused on MM, but since various other hematological tumors and several metastasized solid tumors also exploit the BM to survive and escape from therapy, our data can also be relevant for the response of these tumors to NK cell therapy. We used a series of established MM cell lines to investigate whether hypoxia is an inhibitory factor for effective NK cell anti-tumor responses. In a future study, it would be interesting to understand the influence of hypoxia on the interplay of tumor cells, NK cells and bone marrow stromal cells. Such studies with patient primary MM cells combined with our current knowledge on hypoxia restraining effect on primary NK cells would provide new insights on improving NK cell therapy.

To explore the mechanism of hypoxia mediated down regulation of NK cell cytotoxicity, we investigated, in parallel to NK cell cytotoxicity, the effect of hypoxia on activating- and inhibitory NK cell receptor expression. We observed that hypoxia lowered surface expression of NKG2D and CD16 on NK cells. Previously, hypoxia has been shown to increase tumor cell secretion of MICA that, upon binding, provoked a decrease in NKG2D surface expression [19], [20]. In our experimental set up it seems however unlikely that the decrease in NKG2D expression could be explained through hypoxia induced MICA secretion by the tumor cells. The reduction in NKG2D was namely observed in the absence of MM cells and could be reversed by placing hypoxic NK cells at 21% O_2_. Parkhurst *et al.* have recently reported less functional circulating NK cells, with lower expression of NKG2D and CD16 in melanoma and renal cell carcinoma patients post autologous NK cell infusion [34]. Also in breast cancer patients, decreased expression of activating receptors on NK cells isolated from the tumor has been reported [35]. These patient studies illustrate that the tumor environment can modulate the NK cell phenotype. Although a future study is required to unravel the underlying mechanism, our results provide direct evidence that hypoxia is a tumor environmental factor contributing to the down regulation of key-activating receptors like NKG2D.

In agreement with a previous study [2], we showed that NKG2D is important for *in vitro* killing of MM targets since blocking of this receptor reduced NK cell killing of OPM-1. However, the hypoxia-induced decrease in NKG2D expression was small and our kinetics studies revealed that at least 4 hours of hypoxia were required to reduce surface expression levels. Furthermore, we demonstrated that the oxygen concentration during the killing process was the limiting and critical factor, indicating that the effect was rapid. Together this suggests that NKG2D was probably not the major factor explaining the decrease in cytotoxicity in our experimental model. We also observed a small reduction in CD16 expression on hypoxia incubated NK cells. CD16 is important for antibody mediated cytotoxicity of NK cells. Since our experimental set up did not include MM specific antibodies, it seems unlikely that CD16 is mediating the decrease in cytotoxicity.

Another inhibitory factor could be the secretion of soluble molecules. In MM patients, MICA shedding has been associated with disease progression [36]. Furthermore, interfering with the hypoxia-induced accumulation of HIF-1α and ADAM10 has been shown to enhance MICA surface expression and to reduce MICA shedding, leading to augmented cytotoxicity of PBL against DU145 prostate cancer cells under hypoxia [19]. In our study, we did not observe a change in surface expression of MICA/B or ULBP1-2 under hypoxia on any of the three MM cell lines we tested while CAIX and GLUT1, surrogate markers for hypoxia, were elevated on OPM-1 at 0% O_2_. These markers were not clearly enhanced by hypoxia on RPMI-8226/S and L-363 which might result from differences in regulation or sensitivity between the cell lines. We did not rule out that MICs and ULBPs were shed while maintaining a stable surface expression on the tumor cell. But, addition of supernatant obtained from hypoxic OPM-1 cells to 21% O_2_ cultures did not clearly influence NK cell cytotoxicity in our system (data not shown). This suggested that these soluble mediators were not the major factor for the NK cell response against OPM-1. The inhibitory effect of shedding of soluble NKG2D ligands in response to hypoxia is mainly known for MICA. Since OPM-1 expressed low levels of MICA, it is possible that, in contrast to the prostate cancer cells, MICA is not shed by OPM-1 cells. This illustrates that the secretion of soluble NKG2D ligands is cell type specific. Hence, to conclude that hypoxia induced soluble MICA does not play a role in MM in general, a cell line with higher surface expression of MICA will be required.

To investigate whether deficient degranulation could explain the decreased cytotoxicty under hypoxia, we used an experimental set up that was comparable to the setup of our cytotoxicity assay. In this set up we observed relatively low percentages of CD107a positive NK cells as compared to a previous study by Alter et al. This might be explained by the shorter incubation time that we used (4.5 hours) or by the 16 hours pre-incubation of the NK cells. Nevertheless, we provide evidence that NK cells were able to degranulate in response to OPM-1 cells under hypoxia which indicated that triggering and activation of the NK cells was not completely abolished under hypoxia. However, we did observe a small but significant decrease in the percentage of degranulating NK cells and in the amount of intracellular perforin and granzyme B. The decrease in degranulation was not caused by an intrinsic defect of NK cells to degranulate under hypoxia as incubation of NK cells with a chemical positive control, PMA/ionomycin, did yield comparable levels of degranulation at 21% and 0% O_2_. In response to the universal positive control cell line K562 we did detect decreased NK cell degranulation at 0% O_2_ as compared to 21% O_2_. Therefore, it seems likely that the reduction in degranulation occurred in a target cell line dependent manner. In a previous study, CD8 T cell mediated killing of tumor cells has been shown to be impaired by hypoxia [37]. These authors confirmed that the cytotoxic machinery of CD8 T cells was functional at low oxygen levels and that the decrease in killing could be explained by HIF-1α mediated resistance of the tumor cells. Since we showed that no long-term adaptation of NK- or MM cells was required, it will be interesting to study the contribution of the factors described for CD8 T cells to NK cell anti-tumor responses in an hypoxic environment.

Hypoxia can contribute to the development of chemoresistance. Incomplete chemotherapeutic elimination of tumor cells can be detrimental for the patient and could lead to disease relapse [38]. We recently showed in a mouse model that alloreactive NK cells can cure mice from established 4T1 breast cancer and that this includes the eradication of the population of chemo resistant cells [39]. It would be interesting to investigate whether a similar strategy could help to eliminate chemo resistant MM cells. Alloreactive (KIR-HLA mismatched) NK cells will probably represent the most potent source of NK cells as these subsets will receive a minimal amount of inhibitory signals due to the KIR-HLA mismatch. Alternatively anti-KIR antibodies [8] could help creating a similar effect. Moreover, targeting residual tumor cells within their hypoxic niche with appropriately activated NK cells can be an approach to eradicate tumors.

Together, our data strongly advocates performing any further *in vitro* investigation to study interactions between immune cells and hematological tumor cells - at least MM – also under hypoxic conditions. Furthermore, our study provides proof of concept that activating cytokines can overcome the adverse effects of hypoxia *in vitro* and shows that hypoxia is a factor to take into account when designing allogeneic NK cell based immunotherapy for MM. Experimental set ups comparable to ours will be helpful to determine the potential of novel and existing cytokines or immunomodulatory agents, to boost NK cell responses in a way that NK cells can also exert their effector function in the presence of tumor environmental factors like hypoxia. For future clinical perspectives it might be considered to combine NK cell therapy with hypoxia-targeting and pre-activation of NK cells to eliminate tumor cells in a hypoxic environment.

## Supporting Information

Figure S1 **NK cytotoxicity against OPM-1 is decreased in an hypoxic environment at E:T ratios of 5∶1 and 10∶1.** Both multiple myeloma and NK cells were pre-incubated at different oxygen concentrations and combined in E:T ratios of (A) 5∶1 and (B) 10∶1 in 4.5 hour kill assay. Cytotoxicity was estimated by flow cytometry. Statistics in the figure were performed as: *p<0.05 with one-way repeated measures ANOVA with Bonferroni correction. Each dot represents mean of triplicate cultures for independent donors (*N* = 5).
(PDF)

Figure S2 **Hypoxia does not increase cell death of NK and OPM-1 cells.** (A) Viability of NK cells under different oxygen concentrations was estimated. NK cells were gated on FSC vs SSC to exclude debris. Percentage of early and late apoptotic cells was estimated by the sum of single 7-AAD+, single Annexin V+ and double 7-AAD+Annexin V+ cells. (B) Percentage of viable NK cells was determined as 100 - % (single 7-AAD+, single Annexin V+ and double 7-AAD+Annexin V+ cells) (*N* = 5). (C) OPM-1 cells were incubated for 16 hours at 21% or 0% O_2_ followed by Annexin V- 7AAD apoptosis staining. (D) Spontaneous cell death of OPM-1 as estimated by propidium iodide in independent kill assays consistently ranged between ∼ 6-10% (*N* = 13).
(PDF)

Figure S3 **Flowcytometric analysis of NK cell receptors at 21% and 0% Oxygen.** Flow cytometric analysis of surface expression of NK cell receptors after 14–16 hours of incubation at 21% or 0% O_2_. (A) MACS sorted NK cells were analyzed by flowcytometry. Cells with FSC^high^ were selected for analysis. These cells were >90% pure for CD56 (bottom figure). This population was downstream analyzed for NK receptors in figure B. (B) Histogram plots of NK cell receptors. In each plot, isotype controls at 21% O_2_ (black bold) or 0% O_2_ (black dotted) have been plotted against the respective receptor at 21% O_2_ (green) or 0% O_2_ (red). The percentage of NK cells positive for KIR2DL1, KIR2DL2/3, KIR3DL1, NKG2A and NKG2C are shown against relevant isotype control. The mean fluorescence intensity of the NK cell receptors NKp30, NKp46, NKp80, DNAM-1, LFA-1 and 2B4 have been described in figure 2. The data shown is complete analysis for one individual donor.
(PDF)

Figure S4 **Flowcytometric analysis of intracellular Perforin and Granzyme B at 21% and 0% Oxygen.** Flow cytometric analysis of intracellular Perforin and Granzyme B expression within NK cells after 14–16 hours of incubation at 21% or 0% O_2_. MACS sorted NK cells were analyzed by flowcytometry. The cells were >90% pure for CD56. This population was downstream analyzed for Perforin and Granzyme B expression. Histogram plots representing isotype controls at 21% O_2_ (black bold) or 0% O_2_ (black dotted) have been plotted against the respective intracellular contents at 21% O_2_ (green) or 0% O_2_ (red).
(PDF)

Figure S5 **Percentage of degranulating NK cells is decreased hypoxia.** NK cells and target cells (OPM-1 or K562) were pre-incubated for 14–16 hours at 21% or 0% of oxygen. After this, they were combined in a 4.5 hour degranulation assay in the presence of CD107a. To some of the cultures PMA/ionomycin was added during the 4.5 hour degranulation assay. Data shown here represent paired analysis of CD107a expression on NK cells, in response to (A) OPM-1 (B) PMA/ionomycin and (C) K562. The data represented in this figure are the same data as depicted in figure 5. Each dot is the mean % of CD107a of duplicate cultures of one donor. Lines connect data obtained at 21% and 0% of oxygen for one donor.
(PDF)

Figure S6 **IL-2 activation increases CD107a expression on NK cells both 21% and 0% oxygen.** NK- and OPM-1 MM cells were pre-incubated for 14–16 hours at 21% or 0% of oxygen. During this 14–16 hours, NK cells were either plated in complete media as described in methods and materials, or supplemented additionally with 1000 IU/ml of IL-2. Upon pre-incubation NK cells and MM cells were cocultured in a 4.5 hour CD107a degranulation assay. Degranulation assay was performed either at 21% or 0% of oxygen. Dot plots in this figure are representative of complete analysis for one individual donor for the phenomenon described in figure 6B.
(PDF)

Table S1 **List of antibodies.**
(PDF)
