# Supplementary figures and images for: Hypoxia Induced Impairment of NK Cell Cytotoxicity against Multiple Myeloma Can Be Overcome by IL-2 Activation of the NK Cells
Source: PLoS One. 2013 May 28;8(5):e64835. doi: 10.1371/journal.pone.0064835 (PMC3665801; doi:10.1371/journal.pone.0064835)

Figure S1

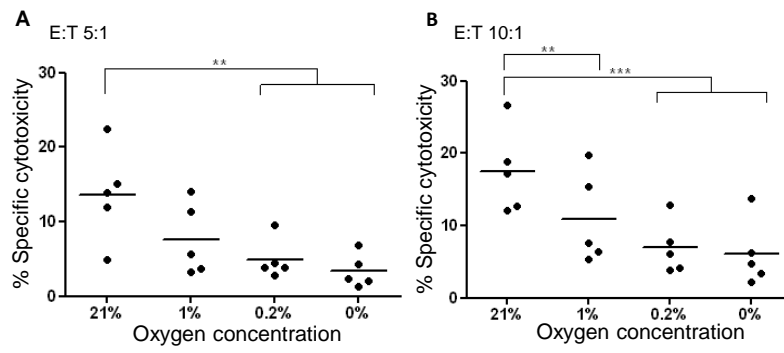

Supplement: Figure S1 — NK cytotoxicity against OPM-1 is decreased in an hypoxic environment at E:T ratios of 5∶1 and 10∶1. Both multiple myeloma and NK cells were pre-incubated at different oxygen concentrations and combined in E:T ratios of (A) 5∶1 and (B) 10∶1 in 4.5 hour kill assay. Cytotoxicity was estimated by flow cytometry. Statistics in the figure were performed as: *p<0.05 with one-way repeated measures ANOVA with Bonferroni correction. Each dot represents mean of triplicate cultures for independent donors (N = 5). (PDF) [file pone.0064835.s001.pdf]

Figure S2

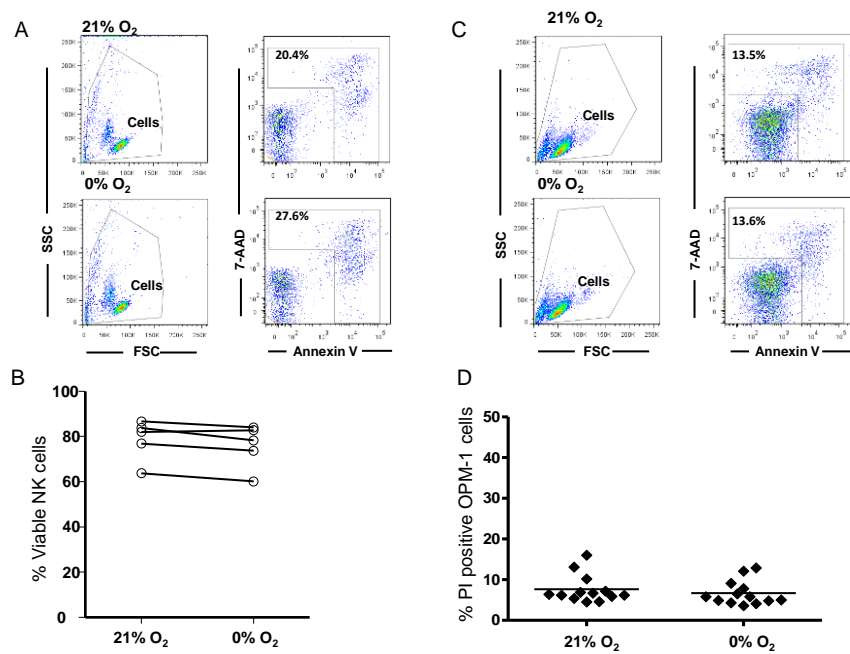

Supplement: Figure S2 — Hypoxia does not increase cell death of NK and OPM-1 cells. (A) Viability of NK cells under different oxygen concentrations was estimated. NK cells were gated on FSC vs SSC to exclude debris. Percentage of early and late apoptotic cells was estimated by the sum of single 7-AAD+, single Annexin V+ and double 7-AAD+Annexin V+ cells. (B) Percentage of viable NK cells was determined as 100 - % (single 7-AAD+, single Annexin V+ and double 7-AAD+Annexin V+ cells) (N = 5). (C) OPM-1 cells were incubated for 16 hours at 21% or 0% O2 followed by Annexin V- 7AAD apoptosis staining. (D) Spontaneous cell death of OPM-1 as estimated by propidium iodide in independent kill assays consistently ranged between ∼ 6-10% (N = 13). (PDF) [file pone.0064835.s002.pdf]

Figure S3

**A**

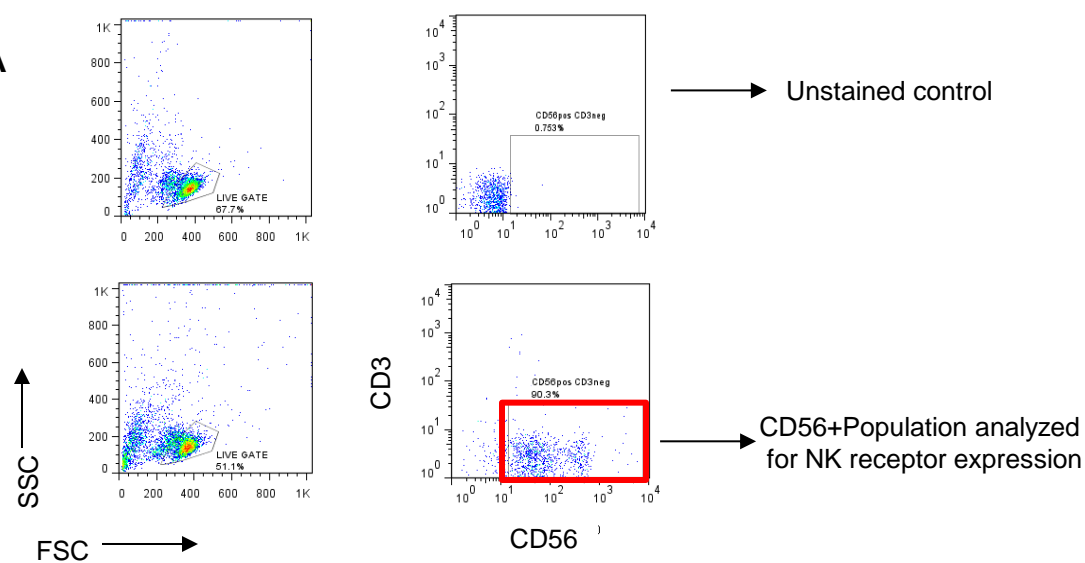

**B**

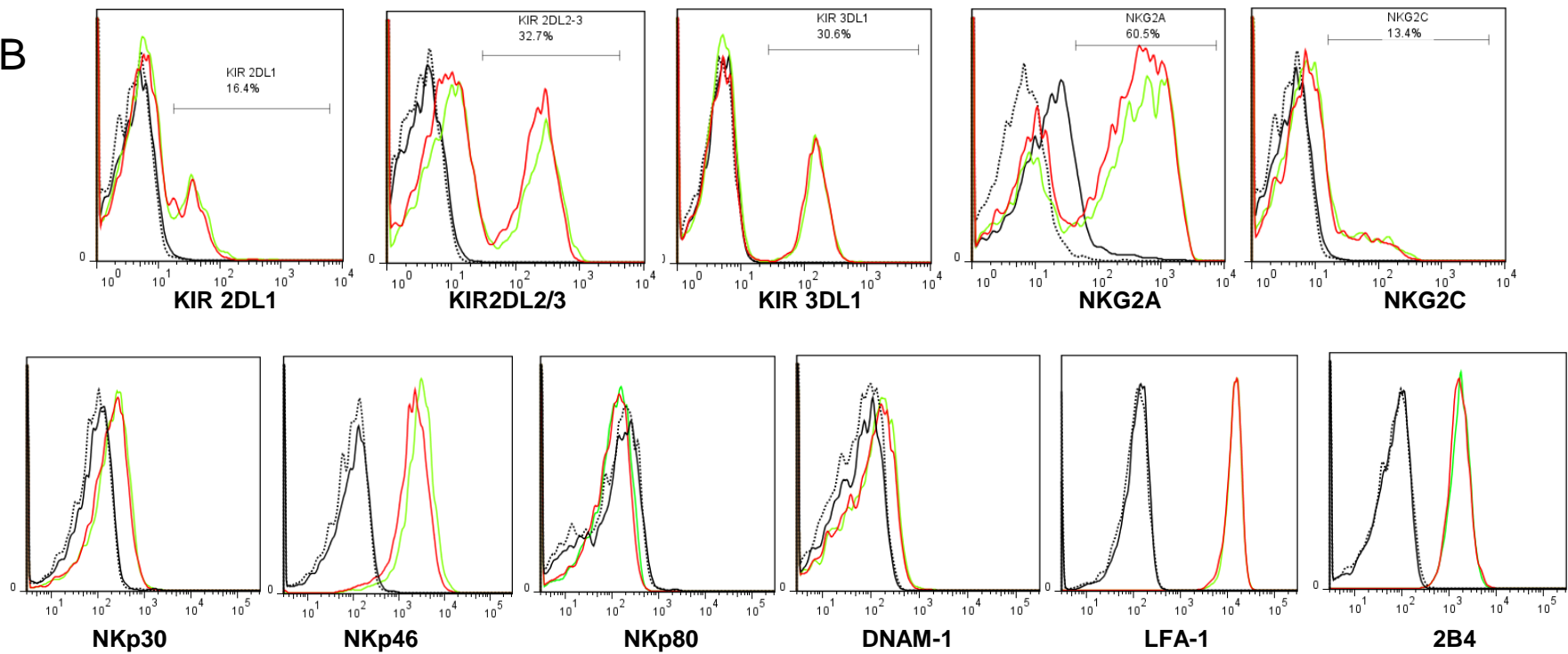

Supplement: Figure S3 — Flowcytometric analysis of NK cell receptors at 21% and 0% Oxygen. Flow cytometric analysis of surface expression of NK cell receptors after 14–16 hours of incubation at 21% or 0% O2. (A) MACS sorted NK cells were analyzed by flowcytometry. Cells with FSChigh were selected for analysis. These cells were >90% pure for CD56 (bottom figure). This population was downstream analyzed for NK receptors in figure B. (B) Histogram plots of NK cell receptors. In each plot, isotype controls at 21% O2 (black bold) or 0% O2 (black dotted) have been plotted against the respective receptor at 21% O2 (green) or 0% O2 (red). The percentage of NK cells positive for KIR2DL1, KIR2DL2/3, KIR3DL1, NKG2A and NKG2C are shown against relevant isotype control. The mean fluorescence intensity of the NK cell receptors NKp30, NKp46, NKp80, DNAM-1, LFA-1 and 2B4 have been described in figure 2. The data shown is complete analysis for one individual donor. (PDF) [file pone.0064835.s003.pdf]

Figure S4

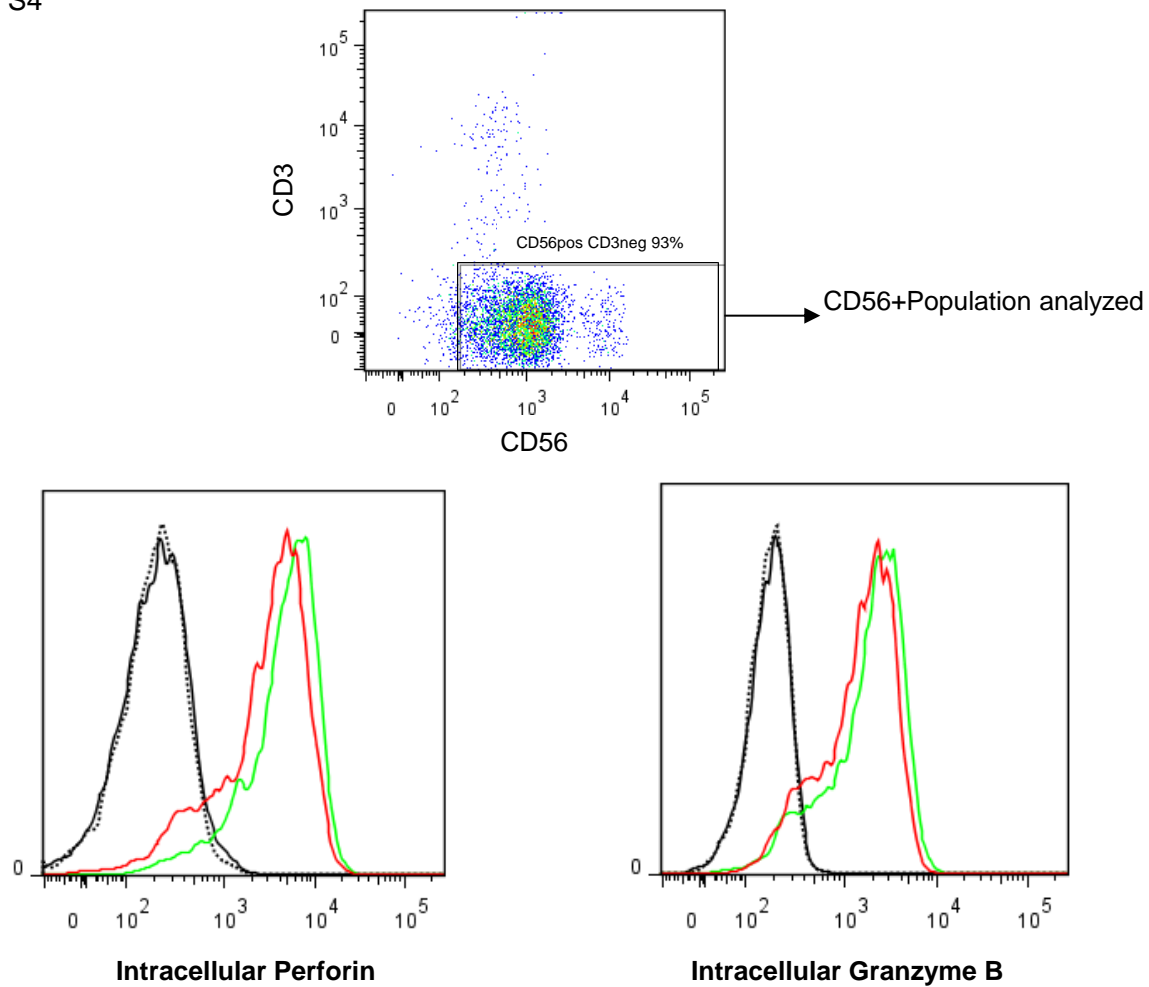

Supplement: Figure S4 — Flowcytometric analysis of intracellular Perforin and Granzyme B at 21% and 0% Oxygen. Flow cytometric analysis of intracellular Perforin and Granzyme B expression within NK cells after 14–16 hours of incubation at 21% or 0% O2. MACS sorted NK cells were analyzed by flowcytometry. The cells were >90% pure for CD56. This population was downstream analyzed for Perforin and Granzyme B expression. Histogram plots representing isotype controls at 21% O2 (black bold) or 0% O2 (black dotted) have been plotted against the respective intracellular contents at 21% O2 (green) or 0% O2 (red). (PDF) [file pone.0064835.s004.pdf]

Figure S5

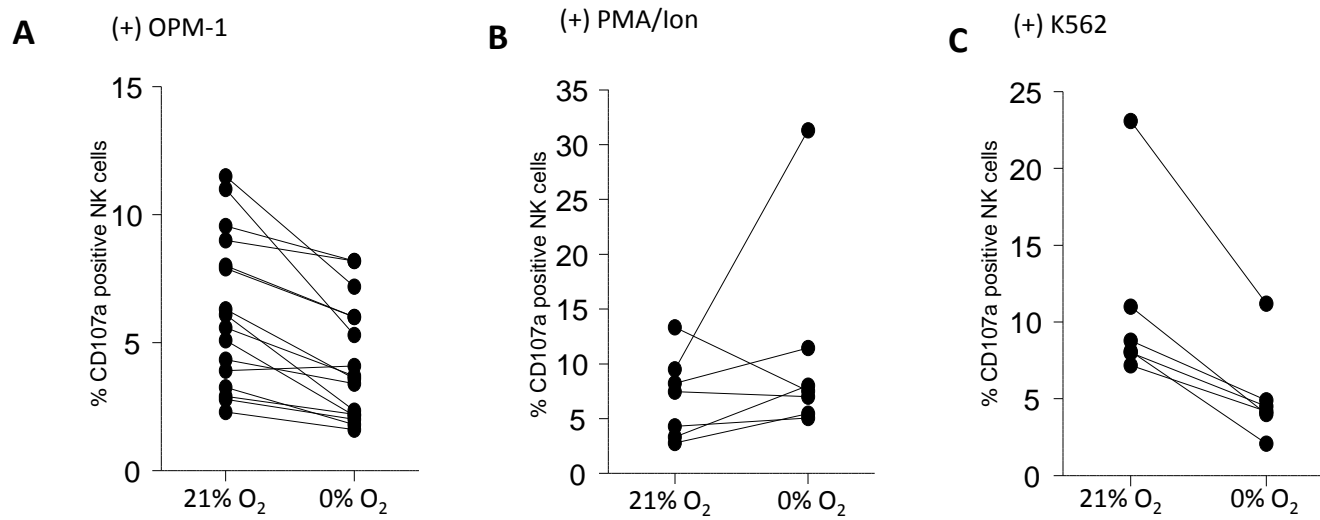

Supplement: Figure S5 — Percentage of degranulating NK cells is decreased hypoxia. NK cells and target cells (OPM-1 or K562) were pre-incubated for 14–16 hours at 21% or 0% of oxygen. After this, they were combined in a 4.5 hour degranulation assay in the presence of CD107a. To some of the cultures PMA/ionomycin was added during the 4.5 hour degranulation assay. Data shown here represent paired analysis of CD107a expression on NK cells, in response to (A) OPM-1 (B) PMA/ionomycin and (C) K562. The data represented in this figure are the same data as depicted in figure 5. Each dot is the mean % of CD107a of duplicate cultures of one donor. Lines connect data obtained at 21% and 0% of oxygen for one donor. (PDF) [file pone.0064835.s005.pdf]

Figure S6

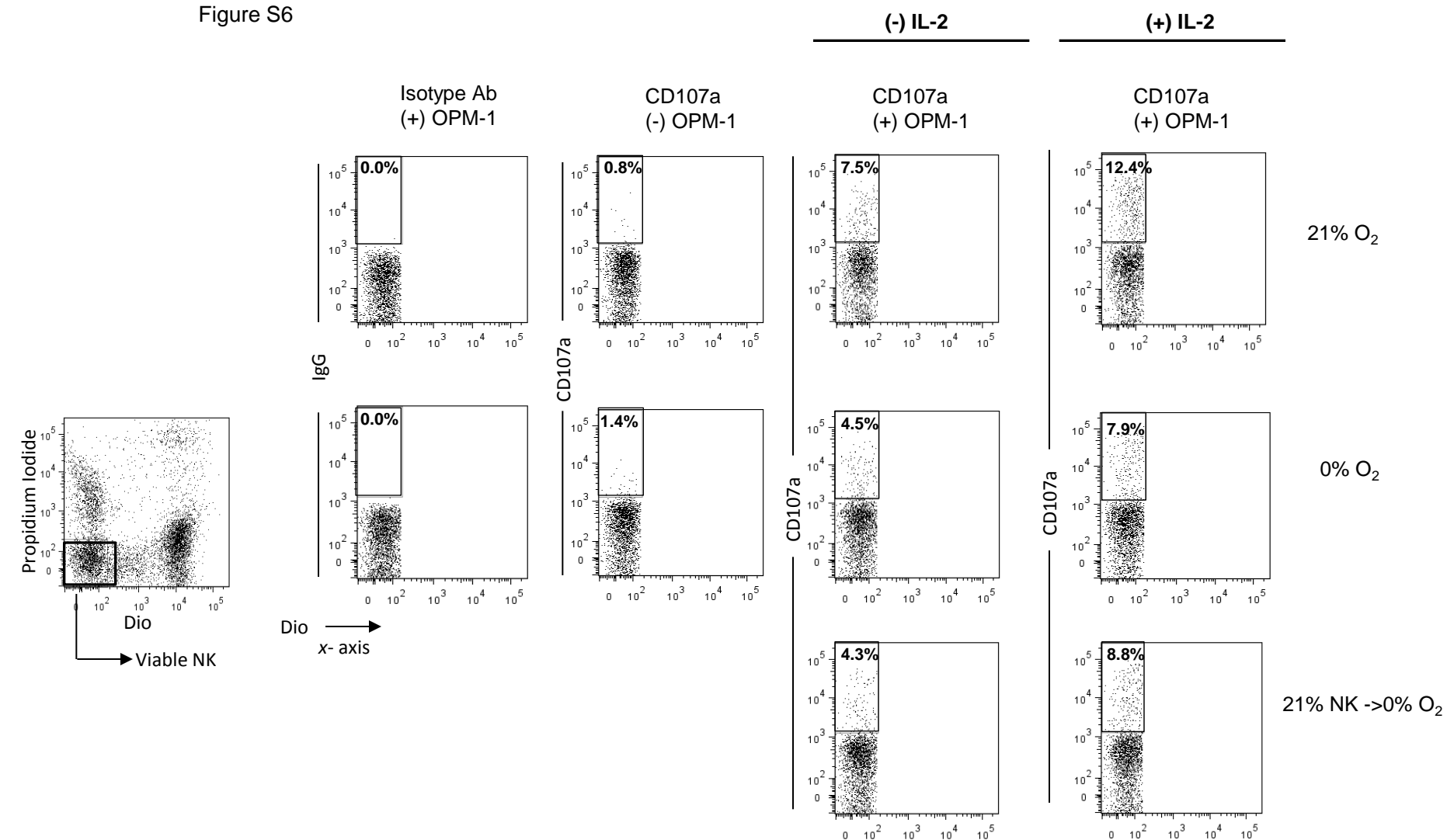

Supplement: Figure S6 — IL-2 activation increases CD107a expression on NK cells both 21% and 0% oxygen. NK- and OPM-1 MM cells were pre-incubated for 14–16 hours at 21% or 0% of oxygen. During this 14–16 hours, NK cells were either plated in complete media as described in methods and materials, or supplemented additionally with 1000 IU/ml of IL-2. Upon pre-incubation NK cells and MM cells were cocultured in a 4.5 hour CD107a degranulation assay. Degranulation assay was performed either at 21% or 0% of oxygen. Dot plots in this figure are representative of complete analysis for one individual donor for the phenomenon described in figure 6B. (PDF) [file pone.0064835.s006.pdf]
