# Supplementary material for: Hypoxia Induced Impairment of NK Cell Cytotoxicity against Multiple Myeloma Can Be Overcome by IL-2 Activation of the NK Cells
Source: PLoS One. 2013 May 28;8(5):e64835. doi: 10.1371/journal.pone.0064835 (PMC3665801; doi:10.1371/journal.pone.0064835)
Supplement: Table S1 — List of antibodies. (PDF) [file pone.0064835.s007.pdf]

Table S1. List of antibodies

Name	Dye	Clone	Company
HLA-E	PE	3D12HLA-E	eBioScience
HLA-ABC	APC	G46-2.6	BD Pharmingen
MICA	PE	159227	R&D SYSTEMS
MICB	APC	2356511	R&D SYSTEMS
ULBP1	PE	170818	R&D SYSTEMS
ULBP2	APC	165903	R&D SYSTEMS
CD3	APC H7	SK7	BD Pharmingen
CD16	FITC	3G8	BD Pharmingen
CD56	Horizon V450	B159	BD Pharmingen
DNAM-1	APC	102511	R&D SYSTEMS
KIR2DL1	PE	143211	R&D SYSTEMS
KIR2DL2/3	APC	DX27	Miltenyi Biotec
KIR3DL1	FITC	DX9	Miltenyi Biotec
LFA1	FITC	HI111	BD Pharmingen
NKG2A	PE-Cy5.5	Z199	Beckman Coultier Inc.
NKG2C	PE	134591	R&D SYSTEMS
NKG2D	APC	BAT221	Miltenyi Biotec
NKp30	PE	210845	R&D SYSTEMS
NKp46	APC	9E2/NKp46	BD Pharmingen
NKp80	APC	239127	R&D SYSTEMS
2B4	PE	Polycl.	R&D SYSTEMS
Perforin	PE	δG9	BD Pharmingen
Granzyme B	FITC	GB11	BD Pharmingen
GLUT1	APC	202915	R&D SYSTEMS
CAIX	FITC	303123	R&D SYSTEMS
